# Association Between Alcohol Consumption and the Risk of Incident Chronic Kidney Disease: A Korean Nationwide Study of Community-Dwelling Older Adults

**DOI:** 10.3390/nu17060983

**Published:** 2025-03-11

**Authors:** In O Sun, Hui-Seung Lee, Chiyeon Lim, Eunjin Bae, Young Youl Hyun, Sungjin Chung, Soon Hyo Kwon, Jang-Hee Cho, Kyung Don Yoo, Woo Yeong Park, Hyunsuk Kim, Byung Chul Yu, Gang-Jee Ko, Jae Won Yang, Won Min Hwang, Sang Heon Song, Sung Joon Shin, Yu Ah Hong

**Affiliations:** 1 Division of Nephrology, Department of Internal Medicine, Presbyterian Medical Center, Jeonju 54987, Republic of Korea; inogood@hanmail.net; 2 Department of Biostatistics, College of Medicine, Dongguk University, Goyang 10326, Republic of Korea; youbil3@naver.com; 3 Department of Radiology, Massachusetts General Hospital and Harvard Medical School, Charlestown, MA 02129, USA; rachun@hanmail.net; 4 Division of Nephrology, Department of Internal Medicine, College of Medicine, Gyeongsang National University, Jinju 52727, Republic of Korea; delight7607@naver.com; 5 Division of Nephrology, Department of Internal Medicine, Kangbuk Samsung Hospital, School of Medicine, Sungkyunkwan University, Seoul 03181, Republic of Korea; femur0@naver.com; 6 Division of Nephrology, Department of Internal Medicine, Yeouido St. Mary’s Hospital, College of Medicine, The Catholic University of Korea, Seoul 07345, Republic of Korea; chungs@catholic.ac.kr; 7 Division of Nephrology, Department of Internal Medicine, Soonchunhyang University Seoul Hospital, Seoul 04401, Republic of Korea; ksoonhyo@schmc.ac.kr; 8 Division of Nephrology, Department of Internal Medicine, Kyungpook National University Hospital, School of Medicine, Kyungpook National University, Daegu 41944, Republic of Korea; jh-cho@knu.ac.kr; 9 Division of Nephrology, Department of Internal Medicine, Ulsan University Hospital, University of Ulsan College of Medicine, Ulsan 44033, Republic of Korea; ykd9062@gmail.com; 10 Division of Nephrology, Department of Internal Medicine, Keimyung University Dongsan Hospital, School of Medicine, Keimyung University, Daegu 42601, Republic of Korea; wy-my@hanmail.net; 11 Division of Nephrology, Department of Internal Medicine, Hallym University Medical Center, Chuncheon Sacred Heart Hospital, Chuncheon 24253, Republic of Korea; keeee@hanmail.net; 12 Division of Nephrology, Department of Internal Medicine, Soonchunhyang University Bucheon Hospital, Bucheon 14584, Republic of Korea; nephroybc@schmc.ac.kr; 13 Division of Nephrology, Department of Internal Medicine, Korea University Guro Hospital, College of Medicine, Korea University, Seoul 08308, Republic of Korea; lovesba@hanmail.net; 14 Division of Nephrology, Department of Internal Medicine, Wonju College of Medicine, Yonsei University, Wonju 26493, Republic of Korea; kidney74@yonsei.ac.kr; 15 Division of Nephrology, Department of Internal Medicine, Konyang University Hospital, Daejeon 35365, Republic of Korea; hwangwm@kyuh.ac.kr; 16 Department of Internal Medicine, Pusan National University School of Medicine, Yangsan 50612, Republic of Korea; shsong0209@gmail.com; 17 Division of Nephrology, Department of Internal Medicine, Dongguk University Ilsan Hospital, School of Medicine, Dongguk University, Goyang 10326, Republic of Korea; shine5050@naver.com; 18 Division of Nephrology, Department of Internal Medicine, Daejeon St. Mary’s Hospital, College of Medicine, The Catholic University of Korea, Seoul 34943, Republic of Korea

**Keywords:** aged, alcohol beverages, chronic kidney disease, glomerular filtration rate, sex

## Abstract

**Background/Objectives:** This study examined the effects of alcohol consumption on chronic kidney disease (CKD) risk in community-dwelling older adults. **Methods:** A nationwide retrospective observational study was conducted using NHIS-Senior cohort data (2009–2018). Adults aged ≥ 65 years with an estimated glomerular filtration rate (eGFR) ≥ 60 mL/min/1.73 m^2^ were included. Alcohol consumption was classified as non-drinking, mild, moderate, or heavy drinking. CKD onset was defined as eGFR < 60 mL/min/1.73 m^2^. **Results:** Of the 122,319 subjects, the non-, mild, moderate, and heavy drinking groups comprised 99,091 (81.0%), 14,842 (12.1%), 4257 (3.5%), and 4139 (3.4%), respectively. During follow-up, 19,796 (20.0%), 4636 (31.2%), 1696 (39.8%), and 1695 (41.0%) patients developed CKD in the non-, mild, moderate, and heavy drinking groups, respectively. Univariate Cox regression analyses showed a significantly increased risk of incident CKD in all drinking groups compared with non-drinkers (all *p* < 0.001). However, hazard ratios (HR) for developing CKD were 0.90 (95% confidence interval [CI] 0.87–0.94, *p* < 0.001) for mild, 0.89 (95% CI 0.84–0.95, *p* < 0.001) for moderate, and 0.93 (95% CI 0.88–0.99, *p* = 0.027) for heavy drinkers. In subgroup analysis, the beneficial effect of alcohol consumption on incident CKD was prominent among moderate drinkers aged 65–74 years and mild drinkers aged ≥ 75 years, in males and mild drinkers aged < 85 years in females. **Conclusions:** This study shows that alcohol consumption is negatively associated with the risk of incident CKD in older adults, particularly among males.

## 1. Introduction

Alcohol consumption is an important global public health issue that causes various social and health problems. Excessive alcohol consumption is known to have harmful effects, such as increasing the risk of liver cirrhosis, pancreatitis, and cancers at various sites [1,2]. In contrast, some epidemiological studies have shown that mild to moderate alcohol consumption reduces the risk of coronary heart disease and type 2 diabetes [3,4]. Therefore, the overall health impact of alcohol consumption remains a topic of ongoing debate, particularly in the context of chronic diseases.

Living a healthy lifestyle is one of the safest and most cost-effective ways to improve one’s quality of life and prevent and/or manage chronic disease. While lifestyle factors, such as physical activity, specific dietary patterns, and smoking, are well-known to be associated with the development of chronic kidney disease (CKD) [5,6,7], the influence of alcohol consumption on CKD development remains inconclusive in the general population [8,9,10,11,12,13,14,15,16,17]. Recent meta-analyses have suggested that alcohol consumption has a beneficial effect on the risk of kidney function decline, particularly in males, regardless of consumption levels [18,19,20]. However, these findings should be interpreted with caution because of variations in sample size, follow-up duration, race, sex of the subjects, and definitions of exposures and outcomes. Given these complexities, further research is needed to better understand the relationship between alcohol consumption and kidney health.

The kidney undergoes structural changes with increasing age, including a decline in the total size and number of nephrons, tubulointerstitial changes, glomerular basement membrane thickening, and increased glomerulosclerosis [21]. These changes contribute to the high susceptibility of the older population to the development of subsequent progressive CKD. Furthermore, while the absorption, metabolism, and excretion of alcohol remain largely unchanged, the consumption of equivalent amounts of alcohol results in elevated blood alcohol concentrations in older population [22]. Therefore, it is common to assume that alcohol consumption negatively impacts kidney health in older adults.

However, information on the association between alcohol consumption and kidney function decline in older adults is limited. To address this gap, we investigated the influence of alcohol intake on the development of CKD in community-dwelling older adults using data from the Senior Cohort Database of the National Health Insurance Service (NHIS-Senior cohort) in Korea.

## 2. Materials and Methods

### 2.1. Study Design and Population

**Figure 1:**
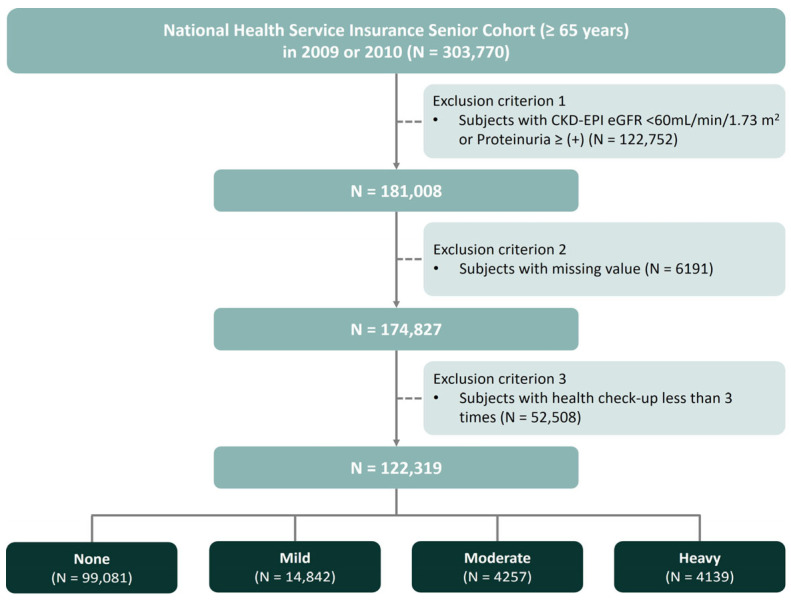
Study design and flow chart for the study participants.

This nationwide, retrospective, observational study was performed using data from the NHIS-Senior cohort between 2009 and 2018, which was established to support research on older adults in Korea [23]. We initially included 303,770 individuals aged ≥ 65 years who underwent a health examination between 2009 and 2010 and had baseline data on alcohol consumption. The cohort was followed up through December 2018 annually to collect anthropometric, sociodemographic, and medical information, including outcomes, and biennially to gather laboratory data from health checkups. Subjects were excluded if they (1) had a baseline estimated glomerular filtration rate (eGFR) < 60 mL/min per 1.73 m^2^ and/or baseline dipstick proteinuria ≥ 1 + (*n* = 122,752), (2) had any missing data (*n* = 6191), or (3) received less than three health screenings during this period (*n* = 52,508). Finally, health screening data was examined from 122,319 participants enrolled in the present study (Figure 1).

This study was conducted in accordance with the guidelines of the Declaration of Helsinki, and all procedures involving research study participants were approved by the Institutional Review Board of the Catholic University of Korea (protocol code: DC22ZISI0019, approval date: 12 April 2022). Informed consent was waived due to the retrospective nature of the study by the National Health Information Data Request Review Committee of the NHIS (NHIS-2023-2-098). Data usage was approved by the National Health Information Data Request Review Committee of the NHIS (NHIS-2023-2-098).

### 2.2. Exposure: Alcohol Intake Patterns

In the self-administered questionnaires, participants were asked about the frequency (number of days per week) and quantity (standard units per occasion) of alcohol consumption. A standard unit was defined as a specialized cup specific to each type of alcohol, such as beer, wine, traditional Korean alcohol (soju), or whiskey. Although different drinks can have very different alcohol content, one standard unit in Korea contains approximately 8 g of ethanol. Daily alcohol intake was assessed based on the total amount of alcohol consumed per week, which was calculated by multiplying the weekly frequency by the amount of pure alcohol consumed per occasion (g of pure alcohol per day). Participants were stratified into four categories based on alcohol consumption: non-drinkers (0 g/day), mild drinkers (<15 g/day), moderate drinkers (15–30 g/day), and heavy drinkers (≥30 g/day).

### 2.3. Baseline Data Collection

Baseline demographic and clinical data were analyzed for all participants. Demographic variables, including age, sex, smoking status, alcohol intake, physical activity, and a history of comorbidities, such as cerebrovascular disease, heart disease, hypertension, diabetes mellitus, and dyslipidemia, were collected from self-reported information. Body mass index (BMI) was calculated by dividing weight (in kilograms) by height squared (in meters), expressed as kg/m^2^. Blood samples were obtained after ≥8-h fasting state and serum creatinine level was measured using an isotope dilution mass spectrometry–calibrated method. Smoking status was categorized as non-smoker, ex-smoker, or current smoker. Regular exercise was defined as ≥3 sessions per week of moderate-intensity exercise lasting >30 min and/or vigorous-intensity exercise for >20 min. The eGFR was calculated using the Chronic Kidney Disease Epidemiology Collaboration (CKD-EPI) equation based on serum creatinine levels [24]. Low income was defined as being in the bottom 20% of National Health Insurance premium payment deciles.

### 2.4. Outcomes

The outcomes were measured using data collected at each visit, even for individuals with longer intervals between visits. The outcome was CKD development, defined as a decrease in eGFR < 60 mL/min per 1.73 m^2^ at any time during the follow-up period in the participants whose baseline eGFR was ≥60 mL/min per 1.73 m^2^.

### 2.5. Statistical Analysis

Continuous variables were expressed as mean and standard deviation, and categorical variables were presented as frequencies with percentages. The Kolmogorov–Smirnov test was used to test whether the variables were normally distributed. *p* values were obtained using the chi-square test for categorical variables. Incidence rates of the development of CKD were calculated as the number of incident cases divided by the entire follow-up period and expressed per 1000 person–years. Hazard ratios (HRs) with 95% confidence intervals (CIs) were obtained using Cox proportional hazards regression analysis. Univariable analyses using Cox proportional hazards regression were performed to determine the risk factors for the development of CKD during follow-up, followed by multivariable analyses to determine significant factors. The linearity assumption for continuous variables was verified using Martingale residual plots. The proportional hazard assumption for categorical variables was verified using a log-minus log plot. Subgroup analyses were conducted based on age, sex, and degree of alcohol intake. All analyses were performed using the R software (version 3.3.3) and SAS version 9.4 (SAS Institute Inc., Cary, NC, USA). Statistical significance was set at *p* < 0.05.

## 3. Results

### 3.1. Characteristics of Participants

The baseline characteristics of the participants, according to alcohol consumption, are summarized in Table 1. The mean age of the total population was 70.5 ± 4.0 years, and 33,707 participants (27.6%) were male. The proportions of participants in each alcohol consumption category were as follows: non-drinkers (81.0%, *n* = 99,091), mild drinkers (12.1%, *n* = 14,842), moderate drinkers (3.5%, *n* = 4257), and heavy drinkers (3.4%, *n* = 4139). Participants in the drinking groups, including mild, moderate, and heavy drinkers, were younger than those in the non-drinking group were. In addition, the proportion of males increased with the level of alcohol consumption. The BMI of the drinking groups was lower than that of the non-drinking group, and individuals in the drinking groups engaged in regular physical exercise more frequently than those in the non-drinking group. Systolic blood pressure (BP) and diastolic BP were higher in the drinking groups than in the non-drinking group. The proportion of ex-smokers and current smokers was higher in the drinking groups than in the non-drinking group. The drinking groups also exhibited a relatively lower prevalence of diabetes mellitus, hypertension, heart disease, cerebrovascular disease, and dyslipidemia compared to the non-drinking group. Baseline creatinine and eGFR levels did not differ between the groups. As alcohol consumption increased, fasting glucose, triglyceride, high-density lipoprotein (HDL) cholesterol, and liver function levels tended to increase, whereas total cholesterol and low-density lipoprotein (LDL) cholesterol levels tended to decrease.

Among males, 49.8% were non-drinkers, 27.2% were mild drinkers, 11.3% were moderate drinkers, and 11.7% were heavy drinkers. Among females, 92.9% were non-drinkers, 6.4% were mild drinkers, 0.5% were moderate drinkers, and 0.2% were heavy drinkers. Baseline characteristics stratified by sex are presented in Supplementary Table S1.

### 3.2. Association Between Alcohol Intake and Risks of Incident Chronic Kidney Disease

During a mean follow-up period of 90.3 ± 23.8 months (range, 36.6–133.7 months), a total of 27,823 (22.7%) patients were diagnosed with CKD (Table 2). Incident CKD was diagnosed in 19,796 (20.0%), 4636 (31.2%), 1696 (39.8%), and 1695 (41.0%) of the non-drinkers, mild drinkers, moderate drinkers, and heavy drinkers, respectively. Overall, the incidence rates of CKD per 1000 person-years were 41.34 in mild drinkers, 52.9 in moderate drinkers, and 55.25 in heavy drinkers, respectively. In terms of sex, the incidence rates of CKD per 1000 person-years were as follows: 56.35 in mild drinkers, 56.5 in moderate drinkers, and 57.23 in heavy drinkers for males; and 17.1 in mild drinkers, 19.8 in moderate drinkers, and 14.2 in heavy drinkers for females.

Next, we analyzed the relative risk of developing CKD during the follow-up period according to alcohol consumption among older people (Table 3). The non-drinking group was used as the reference category to calculate the HRs. In the crude model, the HRs for new-onset CKD were 1.45 (95% CI, 1.50–1.70, *p* < 0.001) for the mild, 1.88 (95% CI, 1.79–1.98, *p* < 0.001) for the moderate, and 2.03 (95% CI, 1.93–2.13, *p* < 0.001) for the heavy groups. In model 1, the lower risk for new-onset CKD was observed only in the mild drinking group (HR 0.92, 95% CI, 0.89–0.95, *p* < 0.001), whereas such finding was found in all drinking groups in Model 2 (Mild: HR 0.90, 95% CI 0.87–0.94, *p* < 0.001, Moderate: HR 0.90, 95% CI 0.85–0.96, *p* = 0.001, Heavy: HR 0.94, 95% CI 0.88–1.00, *p* = 0.04) and Model 3 (Mild: HR 0.90, 95% CI 0.87–0.94, *p* < 0.001, Moderate: HR 0.89, 95% CI 0.84–0.95, *p* < 0.001, Heavy: HR 0.93, 95% CI 0.88–0.99, *p* = 0.027). When analyzed by sex, multivariable Cox regression analysis showed that similar results were found in males (Model 3, Mild: HR 0.92, 95% CI 0.88–0.97, *p* = 0.001; Moderate: HR 0.89, 95% CI 0.84–0.95, *p* < 0.001; Heavy: HR 0.93, 95% CI 0.87–0.99, *p* = 0.031), whereas the protective effect of alcohol intake for CKD development was observed only in females in the mild drinking group (Model 3, HR 0.81, 95% CI 0.74–0.88, *p* < 0.001).

### 3.3. Subgroup Analysis of the Risks of Chronic Kidney Disease Based on Age and Amounts of Alcohol Consumption

**Figure 2:**
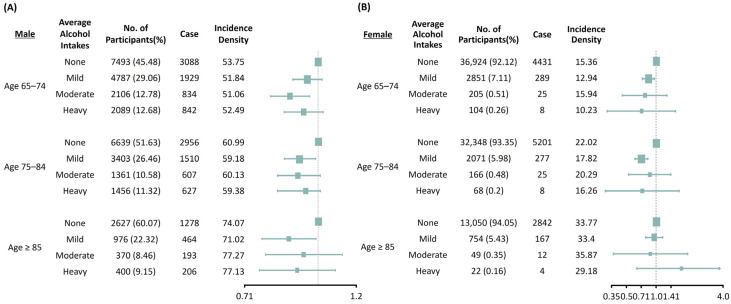
Subgroup analysis of the risks of chronic kidney disease based on age and amounts of alcohol consumption in males (**A**) and females (**B**). Adjusted for age, sex, body mass index, systolic blood pressure, smoking, exercise, diabetes mellitus, hypertension, cerebrovascular disease, heart failure, dyslipidemia, fasting glucose, total cholesterol, low-density lipoprotein cholesterol, alanine aminotransferase, baseline estimated glomerular filtration rate (CKD–EPI).

To investigate the effects of subgroups on the association between alcohol consumption and new-onset CKD, we performed subgroup analyses stratified by age and sex after adjusting for age, sex, comorbidities, BMI, systolic BP, smoking, exercise, diabetes mellitus, hypertension, cerebrovascular disease, heart failure, dyslipidemia, fasting glucose, total cholesterol, low-density lipoprotein cholesterol, alanine aminotransferase and baseline eGFR (Figure 2). In males, the effect of alcohol consumption on the development of CKD was substantially prominent in the moderate drinking group of adults aged 65–74 years (HR 0.87, 95% CI 0.79–0.95). In the participants aged 75–84 years and 85 years and above, only the mild drinking group presented beneficial effects in preventing the development of CKD (age 75–84 years: HR 0.92, 95% CI 0.85–0.99, and age ≥85 years: HR 0.86, 95% CI 0.76–0.98). In females, mild alcohol consumption reduced the development of incident CKD in patients aged 65–74 years (HR 0.84, 95% CI 0.73–0.96) and 75–84 years (HR 0.72, 95% CI 0.63–0.82), but had no significant influence on the development of incident CKD in all subgroups.

## 4. Discussion

This nationwide observational study showed that alcohol consumption was inversely associated with the risk of developing incident CKD in males in all drinking groups and females in the mild drinking group, compared with the non-drinking group, in the Korean community-dwelling older population aged ≥ 65 years. This association was more pronounced among male moderate drinkers aged 65–74 years and mild drinkers aged ≥ 75 years in, and among female mild drinkers aged < 85 years.

The impact of alcohol consumption on the development of CKD has been widely studied in the general population, but studies focusing exclusively on older adults are limited. To date, only two studies have investigated the association between alcohol consumption and kidney function decline in older adults [25,26]. One study demonstrated an inverse linear relationship between moderate alcohol consumption and both the prevalence and incidence of eGFR < 60 mL/min/1.73 m^2^ decline in elderly Italian males aged 65–84 years, but no such relationship was observed in elderly females [25]. In contrast, another community-based longitudinal study found that alcohol consumption had neither adverse nor beneficial effects on rapid kidney function decline in adults aged ≥ 65 years [26]. Our study suggests that alcohol consumption is associated with a reduced risk of incident CKD in older males and that only mild drinking has a protective effect on the development of CKD in older females after adjusting for confounding factors. The strength of this study lies in its analysis of a larger population of older adults using a nationwide Korean observational cohort.

The exact mechanism through which alcohol consumption influences the risk of incident CKD remains unclear. However, some potential mechanisms could explain the favorable effects of alcohol consumption on kidney function. One possible explanation is that moderate alcohol consumption, rather than heavy drinking, may improve lipid metabolism. Several studies have shown that moderate alcohol consumption is associated with an increase in HDL cholesterol levels [27,28,29,30]. Ethanol increases HDL cholesterol by enhancing APOA1 gene expression, which encodes apolipoprotein A-I (ApoA-I) in hepatocytes through RNA polymerase II-mediated transcription [31]. One study also demonstrated that increased alcohol intake is linked to decreased LDL cholesterol and fibrinogen, as well as increased diastolic and systolic BP, and HDL cholesterol [32]. Lipo-toxicity is a well-established risk factor for the development and progression of CKD, and enhanced HDL and reduced LDL levels may help protect against declines in kidney function. At moderate levels, alcohol may have a beneficial effect on lipid metabolism.

Furthermore, alcohol may have antioxidant effects that contribute to its beneficial effects on the kidneys [33]. Oxidative stress plays an important role in the development and progression of CKD [34], and is also implicated in CKD-related complications, particularly cardiovascular complications in CKD patients [35]. One study found that moderate red wine consumption acutely increased plasma total antioxidant capacity and inhibited NF-κB activation induced by a meal, suggesting potential antioxidant benefits [36]. Red wine is known for its high concentration of polyphenolic compounds, and regular and moderate wine consumption may help reduce the incidence of cardiovascular disease and certain types of cancer [37]. Our experimental studies showed that polyphenolic compounds in alcoholic beverages, such as resveratrol, prevent diabetic kidney disease by ameliorating oxidative stress in type 2 diabetic mice [38,39]. Additionally, a low dose of ethanol protects the glomerular filtration barrier through alcohol dehydrogenase (ADH) and 20-hydroxyeicosatetraenoic acid (20-HETE) in podocytes [40]. These studies suggest that low or moderate alcohol consumption may have a protective role in kidney health. In addition, the protective effect on kidney function may vary depending on different types of alcoholic beverages. Further research may be needed to explore the protective effects of different types of alcoholic beverages on kidney health and to determine whether these effects are primarily due to their alcoholic content, such as ethanol, or their non-alcoholic components, such as polyphenols.

Beyond these pathophysiological mechanisms, older adults who consume alcohol may exhibit greater physical activity and overall health compared to non-drinkers. In this study, older adults who consumed alcohol had a higher proportion of regular exercise and lower proportions of comorbidities compared to those who did not consume alcohol. Our recent research also highlighted that high levels of physical activity have beneficial effects in preventing kidney function decline in community-dwelling older adults [41]. Although this study showed that older adults with alcohol consumption have other poor lifestyle habits, such as smoking, smoking as a confounding factor did not affect the relationship between alcohol consumption and kidney function decline. Alcohol consumption may act as a compensatory factor for other lifestyle habits that help lower the risk of CKD in older adults. Further well-designed prospective studies are required to elucidate the precise role of alcohol consumption in kidney function decline among older adults.

When stratified by sex, a negative association between alcohol intake and the development of incident CKD was observed among older males in all drinking groups, whereas it was found only in females with mild drinking. These sex-specific differences in the effects of alcohol consumption on kidney function decline have been documented in previous studies [9,10,12,14]. A recent nationwide cohort study in the general Korean population showed that alcohol consumption had a favorable effect on kidney function exclusively in men [12]. Among older adults, a prior cohort study also revealed an inverse linear relationship between alcohol intake and kidney function decline in males, and a “U-shaped” relationship in females [25]. The possible biological mechanisms underlying these discrepancies remain unclear but may be partially attributed to sex-specific differences in total fluid distribution volume, lean body mass, or liver enzyme activity involved in alcohol metabolism [42,43]. Compared to males, females are more likely to have higher blood alcohol concentrations even when consuming the same amount of alcohol as men. Therefore, alcohol consumption in older females should be approached with caution due to their heightened sensitivity to the effects of alcohol.

Based on the present study, the beneficial effect of alcohol consumption on kidney function decline was obvious in older adults. However, this does not suggest that alcohol consumption is the optimal strategy for the prevention of CKD. The findings of this study should not be interpreted as a recommendation or justification for excessive alcohol consumption to protect kidney function in older adults. Our subgroup analysis demonstrated that the effect of alcohol consumption on CKD incidence varies by age and sex, and the amount of alcohol consumption beneficial for protecting kidney function decreases with age. Excessive alcohol consumption has been associated with several medical, psychological, or social problems. In addition, older adults often take medications that may interact negatively with alcohol or have chronic physical or mental health conditions that could be worsened by its consumption [44]. Some experimental studies have demonstrated that excessive ethanol exposure increases oxidative stress, which could lead to an increase in heart size and negatively affect heart and kidney functions [45,46]. Alcohol-induced oxidative stress is recently reported to be associated with alcoholic neuropathy, which triggers nerve damage through de-escalating the receptors situated in the central nervous system [47]. Therefore, it should be noted that excessive alcohol consumption can have detrimental effects on overall health including kidney in older adults. Given the potential benefits of moderate alcohol intake in protecting kidney function and the associated risks to overall health, alcohol consumption in older adults should be approached with caution and careful consideration.

Our study had several limitations. First, information on alcohol consumption was obtained through self-reported questionnaires, which is likely to have led to underreporting of alcohol intake and may have introduced recall bias. Second, we did not perform detailed analyses based on the type of alcoholic beverage consumed, although several studies have shown that the effects of different types of alcoholic beverages on health are not significant. Moreover, we standardized all beverages to equivalent alcohol content using standard drink units. Third, due to a lack of information, we could not account for changes in the amount or frequency of alcohol consumption over time. Fourth, this study could not investigate whether alcohol consumption primarily affects glomerular or tubulointerstitial lesions as the cause of CKD. Furthermore, the cohort used in this study lacked information on drug use, and the study could not include drug-related confounding factors in CKD development. Further well-designed research is needed to explore the impact of alcohol on kidney function, considering the causes and drug-related confounders of CKD. Lastly, potential residual biases from both measured and unmeasured confounding factors may have influenced the results due to the observational nature of the study. Despite these limitations, to the best of our knowledge, this study is the largest and longest follow-up longitudinal study examining the association between alcohol consumption and CKD incidence in community-dwelling older adults.

## 5. Conclusions

In conclusion, alcohol consumption is negatively associated with the risk of incident CKD in older adults, particularly among males. Future research should focus on the type of alcohol consumed, patterns of drinking habits, changes in alcohol frequency and amount, as well as the combined effects of other lifestyle behaviors on kidney function decline, in order to provide more valuable insights for public health and CKD prevention in older adults.

## Figures and Tables

**Table 1:** Baseline characteristics according to alcohol consumption among 122,319 participants.

		Average Daily Alcohol Consumption			
		None (*n* = 99,081)	Mild (*n* = 14,842)	Moderate (*n* = 4257)	Heavy (*n* = 4139)
Age (year)		70.5 ± 4.0	69.9 ± 3.8	69.6 ± 3.7	69.7 ± 3.6
Age groups (year, n (%))	65–69	44,417 (44.8)	7638 (51.5)	2311 (54.3)	2193 (53.0)
	70–74	38,987 (39.4)	5474 (36.8)	1527 (35.9)	1524 (36.8)
	75–	15,677 (15.8)	1730 (11.7)	419 (9.8)	422 (10.2)
Sex (male, n (%))		16,759 (16.9)	9166 (61.8)	3837 (90.1)	3945 (95.3)
BMI (kg/m^2^)		24.2 ± 9.3	23.8 ± 3.0	23.5 ± 3.0	23.3 ± 2.9
SBP (mmHg)		129.6 ± 15.9	129.8 ± 15.6	131.7 ± 15.7	132.8 ± 16.7
DBP (mmHg)		77.8 ± 9.8	78.3 ± 9.8	79.4 ± 9.8	79.7 ± 10.0
Smoking (n (%))	Non-smoker	88,868 (89.8)	8612 (58.2)	1573 (37.0)	1364 (33.0)
	Ex-smoker	5336 (5.4)	3560 (24.0)	1335 (31.4)	1199 (29.0)
	Current smoker	4770 (4.8)	2637 (17.8)	1339 (31.5)	1573 (38.0)
CVA (n (%))		2310 (3.1)	243 (2.3)	67 (2.2)	62 (2.1)
Heart disease (n (%))		6017 (8.1)	717 (6.8)	194 (6.3)	127 (4.4)
Hypertension (n (%))		42,302 (54.8)	5543 (50.5)	1670 (51.8)	1482 (49.2)
Diabetes (n (%))		13,322 (17.8)	1631 (15.3)	507 (16.2)	484 (16.4)
Dyslipidemia (n (%))		6291 (8.5)	636 (6.0)	164 (5.3)	124 (4.3)
Regular exercise (n (%))		14,253 (14.4)	3131 (21.1)	1039 (24.4)	852 (20.6)
Creatinine (mg/dL)		0.8 ± 0.1	0.8 ± 0.1	0.8 ± 0.1	0.8 ± 0.1
eGFR CKD-EPI (Cr) (mL/min/1.73 m^2^)		80.9 ± 11.1	82.5 ± 9.1	83.5 ± 7.5	83.7 ± 7.2
Fasting glucose (mg/dL)		101.3 ± 24.0	102.1 ± 24.4	104.4 ± 25.5	105.6 ± 26.9
Total cholesterol (mg/dL)		201.1 ± 42.1	195.3 ± 40.4	190.2 ± 35.5	187.9 ± 40.3
Triglyceride (mg/dL)		137.1 ± 86.0	131.5 ± 80.3	142.9 ± 105.9	151.6 ± 115.8
HDL-C (mg/dL)		54.6 ± 29.0	55.9 ± 26.2	58.1 ± 35.2	60.0 ± 39.6
LDL-C (mg/dL)		121.3 ± 52.2	115.2 ± 52.3	107.8 ± 55.6	101.0 ± 45.7
AST (IU/L)		25.4 ± 17.4	26.8 ± 24.3	29.4 ± 19.0	33.6 ± 27.0
ALT (IU/L)		21.9 ± 19.6	23.0 ± 18.5	24.9 ± 17.6	26.6 ± 20.2
Low income, (n (%))	<20%	16,519 (16.7)	2744 (18.5)	765 (18.0)	663 (16.0)

Categorical variables: *p* value obtained from the Chi-square test. Non-categorical variables: *p* value obtained from the Kruskal–Wallis test. BMI, body mass index; SBP, systolic blood pressure; DBP, diastolic blood pressure; CVA, cerebrovascular accident; eGFR, estimated glomerular filtration rate; HDL-C, high-density lipoprotein cholesterol; LDL-C, low-density lipoprotein cholesterol; AST, aspartate aminotransferase; ALT, alanine aminotransferase.

**Table 2:** The development of new-onset CKD according to alcohol consumption in the elderly.

	Observed	Events (%)	Person-Years	Incidence Rates/1000 Person-Years	*p* Value
All					
None	19,796	20.0	732,110.58	27.04	<0.001
Mild	4636	31.2	112,149.39	41.34	
Moderate	1696	39.8	32,059.44	52.90	
Heavy	1695	41.0	30,680.75	55.25	
Male					
None	7322	43.7	123,175.67	59.44	0.216
Mild	3903	42.6	69,260.00	56.35	
Moderate	1634	42.6	28,924.25	56.50	
Heavy	1675	42.5	29,269.61	57.23	
Female					
None	12,474	15.2	608,934.91	20.50	<0.001
Mild	733	13.0	42,889.39	17.10	
Moderate	62	14.8	3135.19	19.80	
Heavy	20	10.3	1411.14	14.20	

**Table 3:** Associations between alcohol intake and risk of new-onset CKD in the elderly.

	Crude				Model 1				Model 2				Model 3			
	HR	95% CI		*p* Value ^1^	HR	95% CI		*p* Value ^1^	HR	95% CI		*p* Value ^1^	HR	95% CI		*p* Value ^1^
		Lower	Upper			Lower	Upper			Lower	Upper			Lower	Upper	
All																
None (Ref)	1				1				1				1			
Mild	1.45	1.40	1.70	<0.001	0.92	0.89	0.95	<0.001	0.90	0.87	0.94	<0.001	0.90	0.87	0.94	<0.001
Moderate	1.88	1.79	1.98	<0.001	0.96	0.91	1.01	0.108	0.90	0.85	0.96	0.001	0.89	0.84	0.95	<0.001
Heavy	2.03	1.93	2.13	<0.001	1.00	0.95	1.05	0.888	0.94	0.88	1.00	0.041	0.93	0.88	0.99	0.027
Male																
None (Ref)	1				1				1				1			
Mild	0.90	0.87	0.94	<0.001	0.936	0.900	0.973	0.001	0.93	0.88	0.97	0.001	0.92	0.88	0.97	0.001
Moderate	0.90	0.85	0.95	<0.001	0.946	0.896	0.998	0.042	0.89	0.84	0.95	0.001	0.89	0.84	0.95	<0.001
Heavy	0.95	0.90	1.00	0.042	0.991	0.939	1.045	0.727	0.94	0.88	1.00	0.047	0.93	0.87	0.99	0.031
Female																
None (Ref)	1				1				1				1			
Mild	0.78	0.72	0.84	<0.001	0.80	0.74	0.86	<0.001	0.79	0.73	0.86	<0.001	0.81	0.74	0.88	<0.001
Moderate	0.96	0.75	1.23	0.721	1.00	0.78	1.28	0.985	0.85	0.62	1.16	0.304	0.85	0.62	1.16	0.306
Heavy	0.69	0.45	1.07	0.097	0.74	0.48	1.15	0.187	0.81	0.50	1.31	0.392	0.87	0.54	1.39	0.553

^1^ *p* value obtained from Cox regression, Model 1: age, sex, Model 2: Model 1 + BMI, SBP, smoking, exercise, diabetes mellitus, hypertension, cerebrovascular disease, heart failure, dyslipidemia, Model 3: Model 2 + fasting glucose, total cholesterol, low-density lipoprotein cholesterol, alanine transaminase, baseline estimated glomerular filtration rate (CKD–EPI).

## Data Availability

Publicly available datasets of Korea were used in this study. These can be found in the NHIS-Senior cohort at https://nhiss.nhis.or.kr assessed on 26 December 2022 [23].

## Supplementary Materials

The following supporting information can be downloaded at: https://www.mdpi.com/article/10.3390/nu17060983/s1, Supplementary Table S1. Baseline characteristics according to alcohol consumption stratified by sex.
