# Supplementary material for: Association Between Alcohol Consumption and the Risk of Incident Chronic Kidney Disease: A Korean Nationwide Study of Community-Dwelling Older Adults
Source: Nutrients. 2025 Mar 11;17(6):983. doi: 10.3390/nu17060983 (PMC11944321; doi:10.3390/nu17060983)
Supplement: Supplementary file 1 [file nutrients-17-00983-s001.zip › nutrients-3501576-supplementary.pdf]

Supplementary Table S1. Baseline characteristics according to alcohol consumption stratified by sex

	Average daily alcohol consumption					Average daily alcohol consumption				
	Male				P-value	Female				P-value
	None	Mild	Moderate	Heavy		None	Mild	Moderate	Heavy	
Age (year)	70.5±4.02	69.76±3.72	69.57±3.63	69.69±3.6	<.0001	70.5±4.02	70.03±3.92	69.92±3.8	69.77±3.84	<.0001
BMI (kg/m^2)	23.2±2.87	23.35±2.76	23.42±2.89	23.24±2.89	<.0001	24.36±10.05	24.41±3.1	24.34±3.36	23.97±3.08	0.044
SBP (mmHg)	128.23±15.47	129.74±15.34	131.75±15.72	132.73±16.57	<.0001	129.89±15.98	129.88±16.1	131.56±16.01	133.39±18.12	0.0022
DBP (mmHg)	77.33±9.68	78.19±9.75	79.37±9.77	79.72±9.98	<.0001	77.89±9.86	78.4±9.95	79.77±9.63	79.99±10.06	<.0001
CVD (n (%))	459(3.89)	161(2.52)	61(2.17)	59(2.13)	<.0001	1851(2.99)	82(1.97)	6(2.06)	3(2.13)	0.0016
Heart disease (n (%))	960(8.09)	435(6.79)	166(5.91)	121(4.37)	<.0001	5057(8.14)	282(6.76)	28(9.56)	6(4.2)	0.0031
Hypertension (n (%))	5235(42.71)	3179(47.94)	1480(50.81)	1399(48.78)	<.0001	37067(57.13)	2364(54.47)	190(61.49)	83(56.85)	0.0025
Diabetes (n (%))	2226(18.61)	1059(16.39)	471(16.63)	470(16.76)	0.0004	11096(17.68)	572(13.61)	36(12.2)	14(9.86)	<.0001
Dyslipidemia (n (%))	490(4.14)	323(5.06)	136(4.84)	119(4.29)	0.0266	5801(9.31)	313(7.48)	28(9.56)	5(3.55)	<.0001
Regular exercise (n (%))	3465(20.68)	2214(24.15)	956(24.92)	824(20.89)	<.0001	10788(13.1)	917(16.16)	83(19.76)	28(14.43)	<.0001
Creatinine (mg/dL)	0.82±0.1	0.82±0.1	0.81±0.1	0.81±0.11	<.0001	0.74±0.12	0.74±0.12	0.73±0.13	0.71±0.13	<.0001
eGFR CKD-EPI (Cr) (mL/min /1.73 m^2)	97.19±48.72	96.7±44.76	97.42±41.91	97.88±37.28	<.0001	80.66±26.57	81.65±23.76	83.14±21.15	85.64±26.54	<.0001
Fasting glucose (mg/dL)	101.88±25.41	102.94±25.45	104.69±25.71	105.72±26.93	<.0001	101.15±23.68	100.83±22.49	102.05±22.92	102.28±24.9	0.2883
Total cholesterol (mg/dL)	186.91±42.09	187.86±40.68	187.87±34.54	186.86±40.19	0.0115	203.96±41.55	207.23±37	211.41±37.43	208.08±37.02	<.0001
Triglyceride (mg/dL)	123.82±79.98	128.65±82.5	142.79±108.53	151.65±116.57	<.0001	139.75±86.93	136.22±76.29	143.57±78.05	151.37±100.18	0.0011
HDL-C (mg/dL)	51.98±30.07	54.74±27.93	57.77±36.71	59.64±37.95	<.0001	55.11±28.77	57.82±23.05	60.85±15.53	66.3±64.33	<.0001
LDL-C (mg/dL)	112.6±49.27	110.2±55.74	105.67±52.74	100.26±46.1	<.0001	123.11±52.54	123.31±45.12	126.72±74.45	115.73±34.44	0.0348
AST (IU/L)	26.11±29.46	27.03±13.83	29.76±19.74	33.52±24.19	<.0001	25.3±13.69	26.44±35.08	26.45±9.94	35.77±60.45	<.0001
ALT (IU/L)	24.11±30.78	23.86±18.05	25.32±18.07	26.6±18.26	<.0001	21.49±16.33	21.7±19	21.34±11.22	26.64±44.2	0.0056

Low income (n, (%))	2926 (17.46)	1702 (18.57)	678 (17.67)	619 (15.69)	0.0011	13593 (16.51)	1042 (18.36)	87 (20.71)	44 (22.68)	<0.0001

Categorical variables: *P* value obtained from the Chi-square test. Non-categorical variables: *P* value obtained from the Kruskal-Wallis test. BMI, body mass index; SBP, systolic blood pressure; DBP, diastolic blood pressure; CVA, cerebrovascular accident; eGFR, estimated glomerular filtration rate; HDL-C, high-density lipoprotein cholesterol; LDL-C, low-density lipoprotein cholesterol; AST, aspartate aminotransferase; ALT, alanine aminotransferase.
